# Short Trips for Short Holidays

**Published:** 1890-09-13

**Authors:** 


### Short Trips for Short Holidays.

### I.—THE NORMAN ARCHIPELAGO.

The grand specimens of Norman architecture in Great Britain will long remain to remind our successors of the landing of William the Conqueror and his companions; but all racial evidence of the invaders may be said to have already vanished from England, save here and there for a few names of men and places. Absorption and dilution have done their work, and it is only in the Channel Islands, as far as English subjects go, that we shall find the Norman race in existence and the Norman-French still spoken. And even here the Anglo-Saxon is slowly but surely displacing the Norman. At the present day the population of the larger towns is more English than Norman, and it is in the country and among the agricultural and fishing population that we look for the old race and the old characteristics. English is spoken by nearly all these; but apparently not as their mother tongue, and it is not very uncommon to find men who speak three languages, namely, English, French, and Norman-French.

The islands all lie in St. Michael's Bay, off the west coast of Normandy, and close to it. Alderney, the nearest to England, is also the nearest to France. It is only sixty miles from the English coast at Portland Bill, and not more than ten from Cape de la Hague, in the department of La Mancha. Those of us who rarely look at maps scarcely realise that Alderney lies nearly due west of the Cape, and, with the exception of this island, all the Channel Islands are further from England than the Cape just mentioned. The whole coast line is very rocky, and rocks of igneous formation are almost the only ones visible, all the stratified rocks having been washed away. Jersey is the furthest south, and is also the largest of the group. St. Helier, with 30,000 inhabitants, is the capital, and in general appearance it does not differ much from an English town of the same size, but when we strike into the country and wander about its lanes, we see the real beauty of the island. Sometimes we drive under an arcade of trees for nearly half a mile, or perhaps a quick turn in the road gives us a glimpse of the clear blue sea, and we literally look down on the lovely coast line, or let our eyes roam over to the shores of France. Around us the signs of life and activity are everywhere in sight. We note the number of cows which even the smallest field seems to keep, and we see that every animal is tethered so that it cannot spoil any of the pasture. Every little corner and square yard of land grows something which can be turned into money. We are told that land rents at from £12 to £16 an acre, and even at this high rental a profit is realised by carefulness and industry. It is true that we see but little evidence of great wealth; and poverty, the too frequent concomitant of wealth is also nowhere to be seen.

Although relatively large sums have been spent on the harbour at St. Helier, it is still impossible for large vessels to enter it at low water. Consequently, it often happens that the steamers have to lie outside, and passengers land in small boats. For this a charge of fivepence or ninepence is made; and, although no one really grudges the islander his little perquisites, there is a general feeling that the fees should be paid by the company which contracts with the passenger to land him in Jersey, or take him away from it. The company's officers answer the traveller by telling him he may embark early, or wait until the tide rises, and enter with the steamer in a certain number of hours; but this is a mere subterfuge. No private individual would dare to make such an excuse, and it is not easy to see why a board should.

Visitors to Jersey rarely omit to explore the Castle of Mont Orgeuil. It is a splendid and almost complete specimen of the old feudal castle and fortress. It is said to date from the reign of Henry II., and there is supposed to be some evidence of Roman occupation. The castle wall is said to have been made by the Romans. Huge chains still hang from various parts of the old battlements, and many a poor wretch's body was suspended from them as a warning to others. In those times the corpses were known as the tassels of the castles. On descending the long flights of steps, one can hardly help reflecting on those so-called good old days and contrasting them with the present ones. Our memory transports us quicker than the fleetest electric current to those mediæval times, and we recall the graceful or terrible legends with which all these old fortresses are enveloped. Then the view from the rock is one of no ordinary beauty. Whichever way we turn the eye rests with pleasure on it. Inland we look at the little farmsteads surrounded with their highly cultivated fields. At our feet the little bay with its fleet of fishing boats, and yonder the coast of La Belle France, with the ever-changing, murmuring sea between. North-east of us and about midway to France, lies a long line of rocks known as the Ecrihous. Here dwells, in a rude hut, a lonely fisherman who calls himself the "Hermit," but others call him the "King." It is a delightful sail to these rocks, and the visitor who invades the old fisherman's kingdom will find his majesty most courteous and intelligent. A packet of newspapers is a very welcome gift.

At Guernsey, the harbour can be entered at all states of the tide, and the chief town, St. Peter Port, is a much more picturesque place than St. Helier. So far we concede the point to the Guersey people, and it is curious to notice how high jealously runs between the islanders; but we do not think the country is so inviting as that of Jersey. While Jersey is the best place from which to make an excursion to Normandy, St. Peter Port is the best starting point for Alderney and Sark and for the islets Herm and Jethou. The distance between Guernsey and Sark can easily be covered within the hour by the little screw steamer that plies weekly or oftener from port to port. We have been accustomed to picture the skippers of steamboats clad in a garb of blue with a favourable eruption of gilt buttons, but our captain affected a certain unconventionalism of attire, and his feet were encased in slippers of a somewhat gaudy hue. "Worked for him, perhaps, by the fair hand of some insular admirer," we ventured to remark *sotto voce*. "Possibly an unhappy attachment," suggested our companion. Nevertheless Monsieur le Capitaine handled his little craft uncommonly well, and brought us alongside the pier as well as any officer in the Royal Navy could have done.

Nowhere in the Channel Islands do the rocks throw themselves into more graceful outlines than around the coast of Sark. The caves and the huge masses of granite which on one hand look like the remains of giant architecture, and on the other are arranged in the most fantastic shapes which set comparison at defiance. The little harbour is itself a wonderful sight; an almost complete amphitheatre of nearly perpendicular rocks rising one hundred feet or more and formed into a perfect haven by a thick granite wall which, like so many of man's works, already shows signs of giving way.

Even if desirable in a paper like this, want of space would prohibit a long description of the islands, and we need do little more than record our opinion that Jersey has the finest drives and walks; Guernsey, the most interesting town; and for beauty of coast line Sark bears away the palm. To the seeker after health or pleasure two or three weeks may well be spent in exploring the little nooks and crannies of these islands, and in many cases a more prolonged residence might be prescribed with advantage.

